# Crystal structure of a new europium(III) compound based on thio­phene­acrylic acid

**DOI:** 10.1107/S2056989022011884

**Published:** 2023-01-01

**Authors:** Suwadee Jiajaroen, Raul Diaz-Torres, Sakchai Laksee, Kittipong Chainok

**Affiliations:** a Thammasat University Research Unit in Multifunctional Crystalline Materials and Applications (TU-MCMA), Faculty of Science and Technology, Thammasat University, Pathum Thani 12121, Thailand; b Nuclear Technology Research and Development Center, Thailand Institute of Nuclear Technology (Public Organization), Nakhon Nayok 26120, Thailand; Universidad Nacional Autónoma de México, México

**Keywords:** crystal structure, europium(III), coordination compound, hydrogen bonds, lanthanide

## Abstract

A europium(III) coordination compound based on thio­phene­acrylic acid (Htpa), tri­aqua­tris­[3-(thio­phen-2-yl)prop-2-enoato-κ^2^ *O*,*O*′]europium(III)–3-(thio­phen-2-yl)prop-2-enoic acid (1/3), [Eu(C_7_H_5_O_2_S)_3_(H_2_O)_3_]·3C_7_H_6_O_2_S or [Eu(tpa)_3_(H_2_O)_3_]·3(Htpa) (**1**), where tpa is the conjugate base of Htpa, has been synthesized and structurally characterized. Compound **1** crystallizes in the trigonal space group *R*3. The structure of **1** consists of a discrete mol­ecular complex [Eu(tpa)_3_(H_2_O)_3_] species and the Htpa mol­ecule. In the crystal, the two components are involved in O—H⋯O [ring motif *R*_2_^2^(8)] and C—H⋯π hydrogen-bonding inter­actions. These inter­actions were further investigated by Hirshfeld surface analysis, which showed high contributions of H⋯H, H⋯C/C⋯H and H⋯O/O⋯H contacts to the total Hirshfeld surfaces.

## Abstract 2

The crystallization and supra­molecular structure of a new europium(III) compound based on thio­phene­acrylic acid is reported.

## 1. Chemical context

In crystal engineering, non-covalent inter­actions are used as a tool in the design and synthesis of functional crystalline materials with predictable structures and desirable physical properties (Desiraju, 2013 ▸; Mirzaei *et al.*, 2014 ▸). Despite the significant number of structures known, this still remains a challenging task, and more especially for the lanthanide-based systems. This is due to the high and variable coordination number exhibited by the 4*f* metals and their small energy difference among various coordination geometries, which can give rise to the appearance of multiple-connected framework structures with a variety of topologies (Sairenji *et al.*, 2016 ▸). In recent years, the design and synthesis of porous materials combining crystal engineering and coordination chemistry have attracted great attention because of their appealing structures and their potential applications in catalysis, ion-exchange, mol­ecular adsorption and chemical sensing (Cawthray *et al.*, 2015 ▸; Pan *et al.*, 2021 ▸; Theppitak *et al.*, 2021 ▸; Jiajaroen *et al.*, 2022 ▸). However, the successful construction of such materials comes only from understanding and controlling the relationship between the geometry frameworks and the involved inter­molecular inter­actions. In this work, we report the synthesis and supra­molecular structure of a new europium(III) compound based on thio­phene­acrylate (tpa), [Eu(tpa)_3_(H_2_O)_3_]·3(Htpa)] (**1**). The inter­molecular inter­actions involved in the formation of the supra­molecular structure of the title compound **1** are discussed in detail. In addition, a Hirshfeld surface analysis was performed to investigate the inter­molecular inter­actions. 
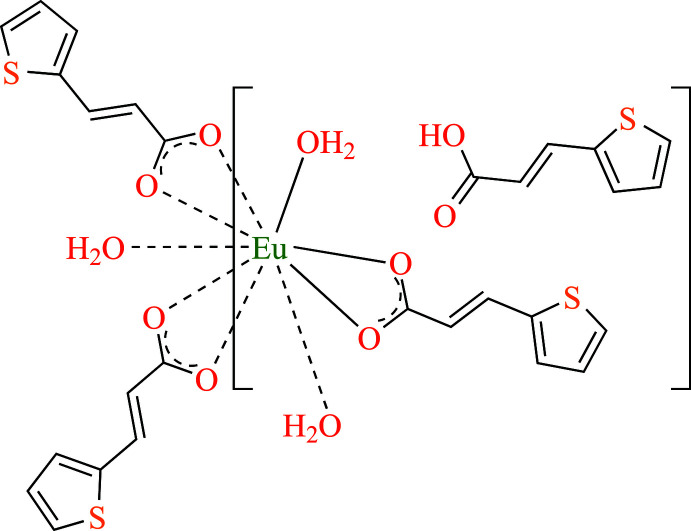


## 2. Structural commentary

**Figure 1:**
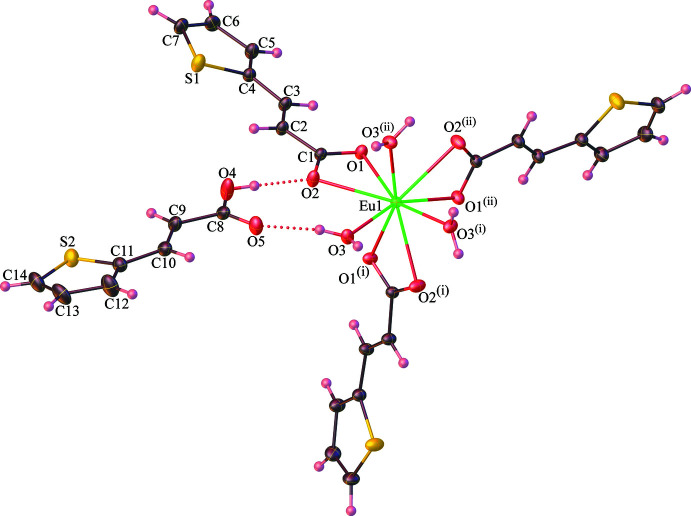
Mol­ecular structure of the title compound **1**. Displacement ellipsoids are drawn at the 30% probability level. All hydrogen atoms were omitted for clarity. Symmetry codes: (i) −*x* + *y*, −*x* + 1, *z*; (ii) −*y* + 1, *x* − *y* + 1, *z*.

**Figure 2:**
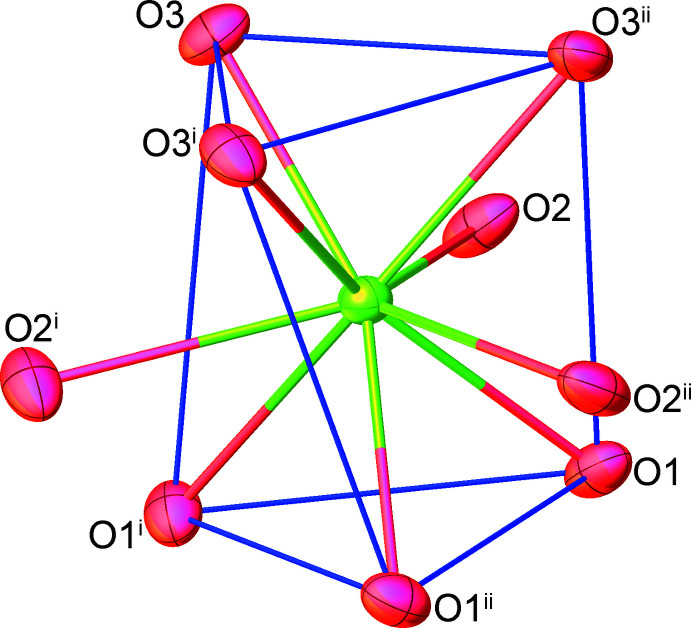
View of the distorted spherical tricapped trigonal prism (TCTPR-9) of the central Eu^III^ ion in the title compound **1**. Symmetry codes: (i) −*x* + *y*, −*x* + 1, *z*; (ii) −*y* + 1, *x* − *y* + 1, *z*.

Single crystal X-ray structural analysis reveals that the title compound **1** crystallizes in the trigonal system with space group *R*3. The Flack parameter (Parsons *et al.*, 2013 ▸) of −0.025 (2) demonstrates the enanti­omeric purity of the tested single crystal. The asymmetric unit consists of one crystallographically independent Eu^III^ ion, one tpa ligand, one Htpa mol­ecule and one coordinated water mol­ecule. As shown in Fig. 1 ▸, the structure of **1** consists of a discrete mol­ecular complex [Eu(tpa)_3_(H_2_O)_3_] and the Htpa mol­ecule. In the discrete complex species, the deprotonated carb­oxy­lic group of tpa ligand adopts a *μ*_1_-κ^2^ *O*,*O′*-chelating coordination mode to the Eu^III^ ion. The central Eu^III^ ion is nine-coordinated with six oxygen atoms from three different tpa ligands and three oxygen atoms from coordinated water mol­ecules. With the assistance of the *SHAPE* program (Llunell *et al.*, 2013 ▸), the coordination geometry around the Eu^III^ center in **1** could be described as a distorted spherical tricapped trigonal prism [TCTPR-9; shape, *D*_3*h*_ symmetry; distortion (τ), 2.761], wherein a trigonal–prismatic geometry is formed by the vertical pairs: O1⋯O3′, O1′⋯O3′′, and O1′′··O3, while the O2, O2′, and O3′′ atoms act as caps as shown in Fig. 2 ▸. The Eu—O bond lengths range from 2.400 (2) to 2.511 (2) Å, and the bond angles range from 51.62 (5) to 157.80 (6)°, which are in the normal ranges of those observed in the reported europium(III) compounds (Behrsing *et al.*, 2016 ▸; Sun *et al.*, 2016 ▸; Alexander *et al.*, 2019 ▸). In addition, the [Eu(tpa)_3_(H_2_O)_3_] complex inter­acts with the Htpa mol­ecule through the formation of an 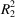 (8) ring motif in terms of graph-set notation (Etter *et al.*, 1990 ▸).

## 3. Supra­molecular features

**Figure 3:**
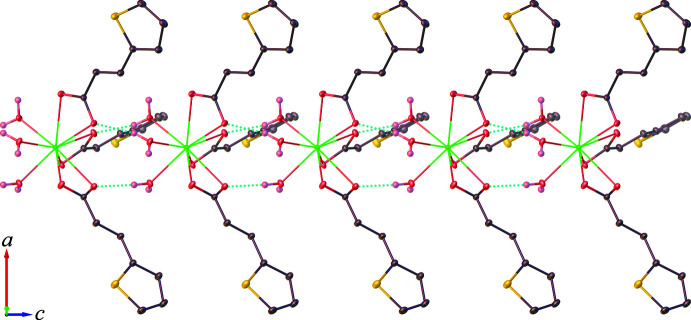
The one-dimensional hydrogen-bonded chain in the title compound **1** running parallel to the *c* axis.

**Figure 4:**
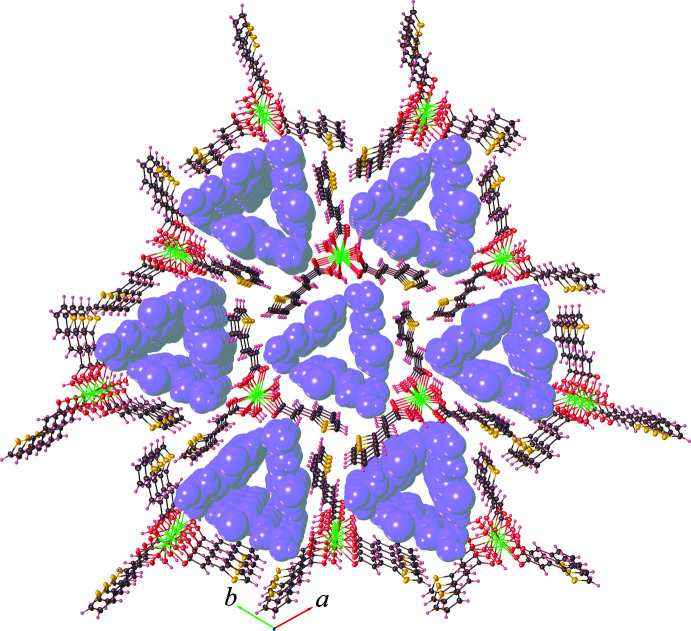
Crystal packing diagram of the title compound **1**, showing the three-dimensional hydrogen-bonded networks of the complex [Eu(tpa)_3_(H_2_O)_3_] species with the Htpa mol­ecules in space-filling representation.

As depicted in Fig. 3 ▸, the discrete complex [Eu(tpa)_3_(H_2_O)_3_] forms a supra­molecular chain extending parallel to the *c* axis with its symmetry-related mol­ecules through classical O—H⋯O hydrogen-bonding inter­actions (Table 1 ▸) between the coordinated water mol­ecules and the carboxyl­ate groups of tpa ligands, which can be described by the 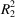 (8) graph-set motif. The chains are further linked *via* C—H⋯π inter­actions involving the thio­phene moieties of adjacent tpa ligands [C7—H7⋯*Cg* distance = 3.869 (3); symmetry code = − 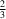 + *y* − *x*, −4/3 − *x*, − 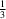 + *z*] . As a result (illustrated in Fig. 4 ▸), a three-dimensional hydrogen-bonded network is created with large channels running along the crystallographic *c-*axis direction. The Htpa mol­ecules are located in the cavities of the network, and are hydrogen bonded to both the tpa and water mol­ecules through inter­molecular O—H⋯O inter­actions with the 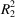 (8) ring motif. It should be noted that no evidence for π–π stacking inter­actions of neighboring aromatic thio­phene rings is observed.

## 4. Hirshfeld surface analysis

**Figure 5:**
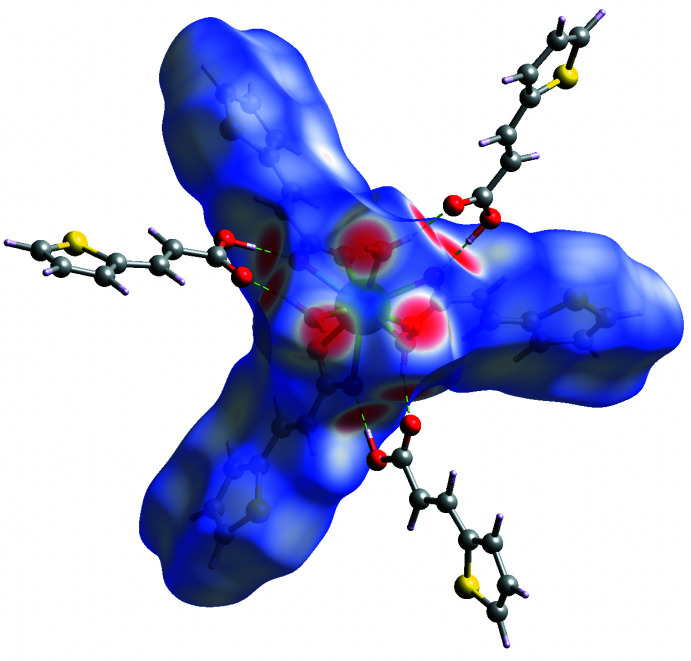
Hirshfeld surface mapped over *d*_norm_ of the title compound **1**, highlighting the O—H⋯O inter­actions.

**Figure 6:**
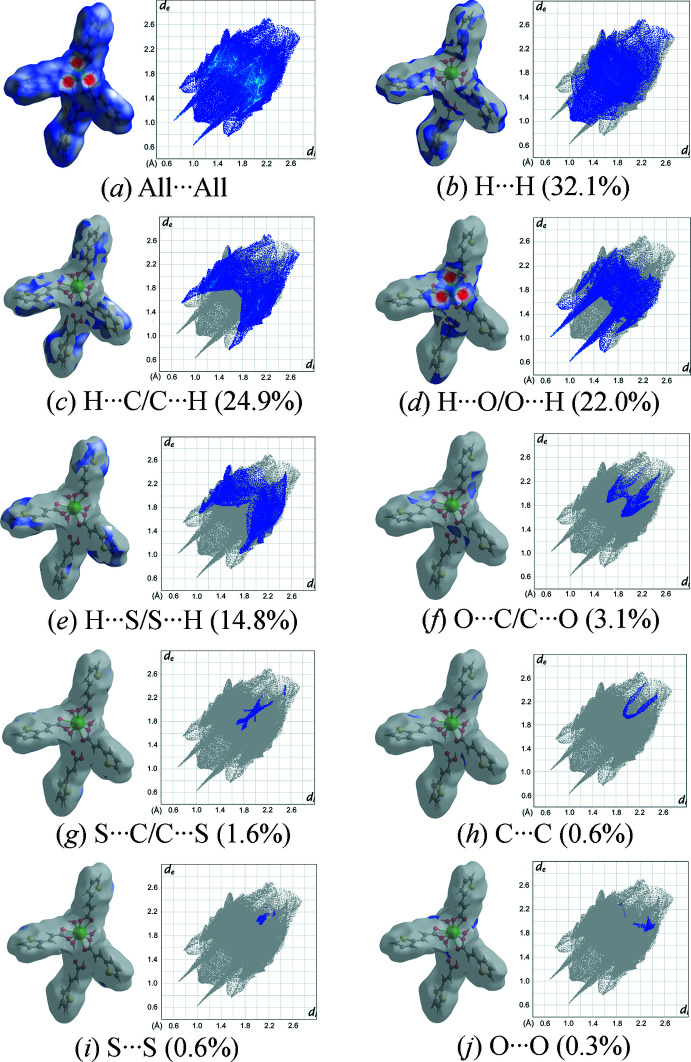
Two-dimensional fingerprint plots of the title compound **1**, showing (*a*) all inter­actions, and those delineated into (*b*) H⋯H, (*c*) H⋯C/C⋯H, (*d*) H⋯O/O⋯H, (*e*) H⋯S/S⋯H, (*f*) O⋯C/C⋯O, (*g*) S⋯C/C⋯S, (*h*) C⋯C, (*i*) S⋯S, and (*j*) O⋯O contacts [*d*_e_ and *d*_i_ represent the distances from a point on the Hirshfeld surface to the nearest atoms outside (external) and inside (inter­nal) the surface, respectively].

The Hirshfeld surfaces and two-dimensional fingerprint plots was generated using *CrystalExplorer 21.5* (Spackman *et al.*, 2021 ▸) in order to qu­antify and visualize the inter­molecular inter­actions in the crystal structure of the title compound **1**. As can be seen in Fig. 5 ▸, the Hirshfeld surfaces mapped over *d*_norm_ shows the most intense red spots around the carboxyl­ate groups and water mol­ecules resulting from the O—H⋯O hydrogen-bonding inter­actions between the complex [Eu(tpa)_3_(H_2_O)_3_] species and the Htpa mol­ecules. Furthermore, analysis of the two-dimensional fingerprint plots, Fig. 6 ▸, reveals that H⋯H (32.1%) contacts, which represent van der Waals inter­actions, are the major contributors toward the Hirshfeld surface. Meanwhile, H⋯C/C⋯H (24.9%, *i.e.* C—H⋯π) and H⋯O/O⋯H (22.0%, *i.e.* O—H⋯O) contacts also make a significant contribution. The H⋯S/S⋯H (14.8%), C⋯O/O⋯C (3.1%) and C⋯S/S⋯C (1.6%) contacts make a small contribution to the entire Hirshfeld surface. Therefore, it can be concluded that O—H⋯O and C—H⋯π hydrogen bonds as well as H⋯H and H⋯S van der Waals contacts contribute significantly to the overall stability of the packing arrangement of the crystal structure of the title compound **1**.

## 5. Infrared spectroscopy

**Figure 7:**
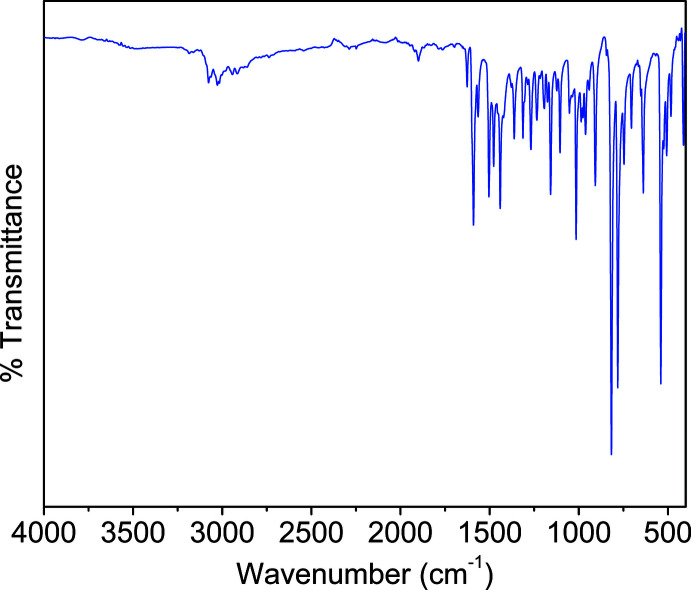
IR spectrum of the title compound **1**.

The infrared (IR) spectrum of the title compound **1** was recorded on a Perkin Elmer model Spectrum 100 spectrometer using the attenuated total reflectance (ATR) mode in the range of 650–4000 cm^−1^. As can be seen in Fig. 7 ▸, the broad absorption bands in the region 3020–3400 cm^−1^ are assigned to the stretching vibrations of the hydroxyl (O—H) groups. The band at 2978 cm^−1^ corresponds to the C—H stretching vibrations. The strong band at 1670 cm^−1^ indicates the existence of the carb­oxy­lic groups while the strong bands appearing in the region 1305–1610 cm^−1^ can be ascribed to the asymmetric and symmetric stretching vibrations of the carboxyl­ate groups. The bands at 705 and 750 cm^−1^ can be assigned to C—S stretching vibrations.

## 6. Thermal stability

**Figure 8:**
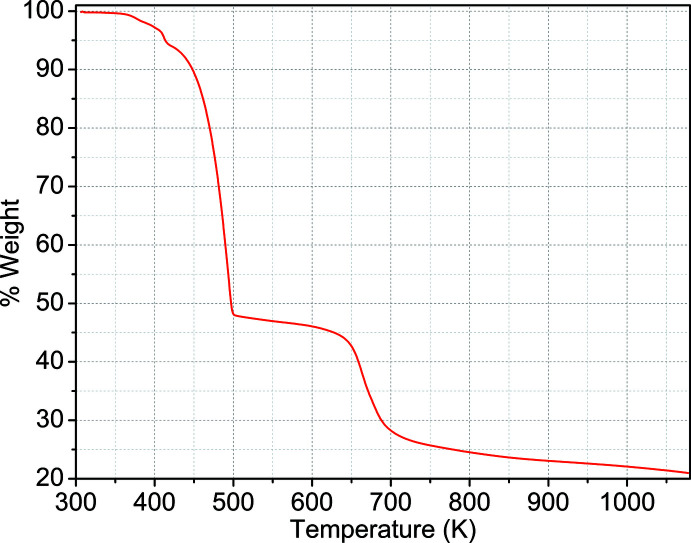
TGA curve of the title compound **1**.

The thermal stability of the title compound **1** was studied by thermogravimetric analysis (TGA). The sample was studied on TGA55 TA Instrument from room temperature to 1073 K under a N_2_ atmosphere (heating rate of 10^o^C min^−1^). As shown in Fig. 8 ▸, the TGA curve of **1** displays two steps of weight loss. The first weight loss of 52.1% from 325–500 K can be ascribed to the removal of water and Htpa mol­ecules (calculated 50.7%). Then the structure begins to collapse at around 630 K.

## 7. Database survey

A ConQuest search for the metal complexes bearing the thio­phene­acrylate ligand in the Cambridge Structural Database (CSD version 5.42, September 2021 update; Bruno *et al.*, 2002 ▸; Groom *et al.*, 2016 ▸) resulted in ten hits, namely, the complexes with the Mo^V^ ion (GAKPUF, Vrdoljak *et al.*, 2010 ▸; DAMRUG, Alberding *et al.*, 2011 ▸), Sb^V^ ion (GIFPET, GIFPIX, Sarwar *et al.*, 2018 ▸), Sn^IV^ ion (NUJGII, Danish *et al.*, 1996 ▸; RIWBII, Parvez *et al.*, 1997 ▸; TEDTIF, TEDTOL, Danish *et al.*, 1995 ▸), Ga^III^ ion (YUWCAV, Uhl *et al.*, 2010 ▸), and Pd^II^ ion (ZIJNAK, Vasseur *et al.*, 2018 ▸). In these complexes, the tpa ligand displays four distinct coordination modes with the carboxyl­ate anions being monodentate *μ*_1_-κ^1^ *O* (GIFPET, GIFPIX), bidentate chelating *μ*_1_-κ^2^ *O*,*O′* (RIWBII, ZIJNAK, similar to that found in the title compound **1**) and *μ*_2_-κ*O*:κ*O* (DAMRUG, GAKPUF, NUJGII, YUWCAV), or bidentate bridging *μ*_2_-κ*O*:κ*O*′ (TEDTIF, TEDTOL). In addition, 69 hits for lanthanide complexes with the [*Ln*(COO)_3_(H_2_O)_3_] coord­ination sphere similar to that in the title compound **1** were found. Twelve of them *viz*. CSD refcodes HIVCEW, HIVCIA, HIVCOG, HIVCUM, HIVDAT (Marques *et al.*, 2013 ▸), LOMNAE (Tsaryuk *et al.*, 2014 ▸), VUSGIZ, VUSGOF, VUSGUL (Zeng & Pan, 1992 ▸), XILLUA (Kameshwar *et al.*, 2007*a* ▸), XILNUC (Kameshwar *et al.*, 2007*b* ▸), and YENHOO (Rzaczynska & Belskii, 1994 ▸) crystallized in the trigonal system with space group *R*3, and the central *Ln* ^3+^ cation exhibiting a nine-coordinated tricapped trigonal–prismatic (TTP) geometry.

## 8. Synthesis and crystallization

All reagents were purchased as analytical grade and used without further purification. The Htpa ligand (30.8 mg, 0.2 mmol) was dissolved in an iso­propanol solution (2 ml) and was then added dropwise to an aqueous solution (5 ml) of Eu(NO_3_)_3_·6H_2_O (44.61 mg, 0.1 mmol). The mixture was stirred for 1 h at room temperature and then filtered to remove any undissolved solid. The solution was slowly evaporated at room temperature. Colorless block-shaped crystals of **1** were obtained in 20% yield (8.9 mg) based on Eu^3+^ source.

## 9. Refinement

Crystal data, data collection and structure refinement details are summarized in Table 2 ▸. All non-hydrogen atoms were refined anisotropically. Hydrogen atoms attached to carbon atoms were refined in the riding-model approximation with C—H = 0.93 Å and *U*_iso_(H) = 1.2*U*_eq_(C). Hydrogen atoms bounded to oxygen atoms of coordinated water (O3) and carb­oxy­lic acid (O4) were located from difference-Fourier maps but were refined with distance restraints of O—H = 0.84 ± 0.01 Å and with *U*_iso_(H) = 1.5*U*_eq_(O). The thio­phene ring of the Htpa mol­ecule was found to be disordered over two positions and the site occupancies of the disordered fragments were refined to 0.778 (4) and 0.222 (4). The restraints of the SADI, RIGU and FLAT commands were applied to accommodate the disordered thio­phene ring.

## Supplementary Material

Crystal structure: contains datablock(s) I. DOI: 10.1107/S2056989022011884/jq2020sup1.cif

Structure factors: contains datablock(s) I. DOI: 10.1107/S2056989022011884/jq2020Isup2.hkl

Supporting information file. DOI: 10.1107/S2056989022011884/jq2020Isup3.cdx

CCDC reference: 2226237

Additional supporting information:  crystallographic information; 3D view; checkCIF report

## Figures and Tables

**Table 1:** Hydrogen-bond geometry (Å, °) *Cg*1 is the centroid of the S1/C4–C7 ring.

*D*—H⋯*A*	*D*—H	H⋯*A*	*D*⋯*A*	*D*—H⋯*A*
O3—H3*A*⋯O5	0.82 (1)	1.94 (2)	2.729 (3)	162 (3)
O3—H3*B*⋯O1^i^	0.82 (1)	1.89 (2)	2.693 (2)	165 (3)
O4—H4⋯O2	0.84 (1)	1.78 (2)	2.614 (3)	177 (4)
C7—H7⋯*Cg*1^ii^	0.93	3.10	3.869 (3)	141

Symmetry codes: (i) 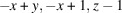 ; (ii) 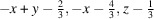 .

**Table 2:** Experimental details

Crystal data	
Chemical formula	[Eu(C_7_H_5_O_2_S)_3_(H_2_O)_3_]·3C_7_H_6_O_2_S
*M*_r_	1128.05
Crystal system, space group	Trigonal, *R*3
Temperature (K)	296
*a*, *c* (Å)	26.5369 (6), 5.9386 (1)
*V* (Å^3^)	3621.72 (17)
*Z*	3
Radiation type	Mo *K*α
μ (mm^−1^)	1.62
Crystal size (mm)	0.28 × 0.22 × 0.12

Data collection	
Diffractometer	Bruker D8 QUEST CMOS
Absorption correction	Multi-scan (*SADABS*; Krause *et al.*, 2015 ▸)
*T*_min_, *T*_max_	0.690, 0.746
No. of measured, independent and observed [*I* > 2σ(*I*)] reflections	54319, 4921, 4921
*R*_int_	0.036
(sin θ/λ)_max_ (Å^−1^)	0.715

Refinement	
*R*[*F* ^2^ > 2σ(*F* ^2^)], *wR*(*F* ^2^), *S*	0.015, 0.032, 1.07
No. of reflections	4921
No. of parameters	252
No. of restraints	88
H-atom treatment	H atoms treated by a mixture of independent and constrained refinement
Δρ_max_, Δρ_min_ (e Å^−3^)	0.21, −0.25
Absolute structure	Flack *x* determined using 2459 quotients [(*I* ^+^)−(*I* ^−^)]/[(*I* ^+^)+(*I* ^−^)] (Parsons *et al.*, 2013 ▸)
Absolute structure parameter	−0.024 (2)

Computer programs: *APEX4* and *SAINT* (Bruker, 2019 ▸), *SHELXT* (Sheldrick, 2015*a* ▸), *SHELXL2018/3* (Sheldrick, 2015*b* ▸), and *OLEX2* (Dolomanov *et al.*, 2009 ▸).

## supplementary crystallographic information

### Crystal data

**Table:**

[Eu(C_7_H_5_O_2_S)_3_(H_2_O)_3_]·3C_7_H_6_O_2_S	*D*_x_ = 1.552 Mg m^−^^3^
*M**_r_* = 1128.05	Mo *K*α radiation, λ = 0.71073 Å
Trigonal, *R*3	Cell parameters from 9889 reflections
*a* = 26.5369 (6) Å	θ = 2.7–30.4°
*c* = 5.9386 (1) Å	µ = 1.62 mm^−^^1^
*V* = 3621.72 (17) Å^3^	*T* = 296 K
*Z* = 3	Block, colourless
*F*(000) = 1710	0.28 × 0.22 × 0.12 mm

### Data collection

**Table:**

Bruker D8 QUEST CMOS diffractometer	4921 independent reflections
Radiation source: sealed x-ray tube, Mo	4921 reflections with *I* > 2σ(*I*)
Graphite monochromator	*R*_int_ = 0.036
Detector resolution: 7.39 pixels mm^-1^	θ_max_ = 30.6°, θ_min_ = 2.7°
φ and ω scans	*h* = −37→37
Absorption correction: multi-scan (SADABS; Krause *et al.*, 2015)	*k* = −37→37
*T*_min_ = 0.690, *T*_max_ = 0.746	*l* = −8→8
54319 measured reflections	

### Refinement

**Table:**

Refinement on *F*^2^	H atoms treated by a mixture of independent and constrained refinement
Least-squares matrix: full	*w* = 1/[σ^2^(*F*_o_^2^) + (0.0145*P*)^2^ + 0.9645*P*] where *P* = (*F*_o_^2^ + 2*F*_c_^2^)/3
*R*[*F*^2^ > 2σ(*F*^2^)] = 0.015	(Δ/σ)_max_ = 0.001
*wR*(*F*^2^) = 0.032	Δρ_max_ = 0.21 e Å^−^^3^
*S* = 1.07	Δρ_min_ = −0.25 e Å^−^^3^
4921 reflections	Extinction correction: SHELXL-2018/3 (Sheldrick, 2015b), Fc^*^=kFc[1+0.001xFc^2^λ^3^/sin(2θ)]^-1/4^
252 parameters	Extinction coefficient: 0.00059 (8)
88 restraints	Absolute structure: Flack *x* determined using 2459 quotients [(*I*^+^)-(*I*^-^)]/[(*I*^+^)+(*I*^-^)] (Parsons *et al.*, 2013)
Hydrogen site location: mixed	Absolute structure parameter: −0.024 (2)

### Special details

**Table:**

Geometry. All esds (except the esd in the dihedral angle between two l.s. planes) are estimated using the full covariance matrix. The cell esds are taken into account individually in the estimation of esds in distances, angles and torsion angles; correlations between esds in cell parameters are only used when they are defined by crystal symmetry. An approximate (isotropic) treatment of cell esds is used for estimating esds involving l.s. planes.

### Fractional atomic coordinates and isotropic or equivalent isotropic displacement parameters (Å^2^)

**Table:**

	*x*	*y*	*z*	*U*_iso_*/*U*_eq_	Occ. (<1)
Eu1	0.333333	0.666667	0.48411 (2)	0.02639 (5)	
S1	0.36988 (4)	0.42204 (3)	0.93748 (13)	0.05608 (18)	
S2	0.07634 (8)	0.25613 (8)	0.0719 (3)	0.0619 (4)	0.778 (4)
S2A	0.0659 (6)	0.3006 (7)	−0.330 (2)	0.075 (2)	0.222 (4)
O1	0.36847 (8)	0.62424 (7)	0.7756 (3)	0.0377 (3)	
O2	0.30981 (8)	0.56296 (7)	0.5258 (3)	0.0445 (4)	
O3	0.26557 (8)	0.60435 (7)	0.2042 (3)	0.0391 (3)	
H3A	0.2574 (13)	0.5703 (7)	0.197 (5)	0.031 (7)*	
H3B	0.2612 (13)	0.6169 (12)	0.083 (3)	0.051 (8)*	
O4	0.24635 (12)	0.45449 (9)	0.3997 (5)	0.0819 (8)	
H4	0.2673 (14)	0.4894 (8)	0.436 (6)	0.077 (11)*	
O5	0.22298 (10)	0.48956 (8)	0.1065 (4)	0.0591 (5)	
C1	0.34166 (10)	0.57296 (10)	0.6978 (4)	0.0327 (4)	
C2	0.34785 (11)	0.52549 (10)	0.7943 (4)	0.0405 (5)	
H2	0.330251	0.489999	0.719057	0.049*	
C3	0.37665 (11)	0.52981 (11)	0.9802 (4)	0.0403 (5)	
H3	0.391555	0.564646	1.059115	0.048*	
C4	0.38750 (10)	0.48571 (10)	1.0746 (4)	0.0395 (5)	
C5	0.41575 (12)	0.48962 (11)	1.2707 (4)	0.0477 (6)	
H5	0.428911	0.520927	1.369331	0.057*	
C6	0.42297 (13)	0.44089 (13)	1.3086 (5)	0.0548 (7)	
H6	0.441164	0.436683	1.435195	0.066*	
C7	0.40079 (14)	0.40149 (13)	1.1425 (5)	0.0541 (7)	
H7	0.402122	0.367138	1.139609	0.065*	
C8	0.21768 (11)	0.44876 (11)	0.2140 (5)	0.0476 (6)	
C9	0.17650 (12)	0.38762 (11)	0.1581 (5)	0.0513 (6)	
H9	0.174401	0.358161	0.249464	0.062*	
C10	0.14254 (11)	0.37414 (12)	−0.0201 (5)	0.0496 (6)	
H10	0.148027	0.405028	−0.110983	0.060*	0.778 (4)
H10A	0.146668	0.403635	−0.116266	0.060*	0.222 (4)
C11	0.0978 (3)	0.3170 (2)	−0.0906 (16)	0.050 (2)	0.778 (4)
C11A	0.0989 (6)	0.3144 (9)	−0.070 (5)	0.051 (7)	0.222 (4)
C12	0.0688 (7)	0.3046 (7)	−0.282 (2)	0.080 (3)	0.778 (4)
H12	0.072978	0.332514	−0.386972	0.097*	0.778 (4)
C12A	0.0791 (12)	0.2676 (11)	0.049 (5)	0.085 (11)	0.222 (4)
H12A	0.092117	0.267347	0.194105	0.102*	0.222 (4)
C13	0.0297 (3)	0.2428 (3)	−0.3118 (14)	0.080 (2)	0.778 (4)
H13	0.007440	0.226422	−0.440497	0.095*	0.778 (4)
C13A	0.0345 (12)	0.2148 (11)	−0.063 (4)	0.063 (6)	0.222 (4)
H13A	0.016090	0.177634	−0.001909	0.076*	0.222 (4)
C14	0.0292 (4)	0.2116 (4)	−0.1301 (15)	0.073 (2)	0.778 (4)
H14	0.006362	0.171352	−0.117203	0.087*	0.778 (4)
C14A	0.0245 (10)	0.2287 (9)	−0.269 (5)	0.075 (8)	0.222 (4)
H14A	−0.002560	0.201670	−0.368413	0.090*	0.222 (4)

### Atomic displacement parameters (Å^2^)

**Table:**

	*U*^11^	*U*^22^	*U*^33^	*U*^12^	*U*^13^	*U*^23^
Eu1	0.02863 (5)	0.02863 (5)	0.02191 (6)	0.01432 (3)	0.000	0.000
S1	0.0759 (5)	0.0441 (3)	0.0540 (4)	0.0343 (3)	−0.0218 (3)	−0.0081 (3)
S2	0.0656 (9)	0.0470 (7)	0.0633 (8)	0.0208 (6)	−0.0086 (6)	−0.0082 (5)
S2A	0.067 (4)	0.078 (4)	0.069 (5)	0.029 (3)	−0.032 (3)	−0.025 (3)
O1	0.0486 (10)	0.0343 (8)	0.0331 (8)	0.0229 (7)	−0.0127 (7)	−0.0072 (6)
O2	0.0589 (10)	0.0349 (8)	0.0390 (8)	0.0228 (8)	−0.0222 (7)	−0.0056 (6)
O3	0.0454 (9)	0.0355 (9)	0.0316 (8)	0.0168 (8)	−0.0122 (7)	0.0017 (7)
O4	0.0987 (18)	0.0395 (11)	0.0989 (19)	0.0282 (12)	−0.0581 (15)	−0.0183 (11)
O5	0.0659 (13)	0.0417 (10)	0.0584 (12)	0.0184 (9)	−0.0173 (10)	−0.0091 (9)
C1	0.0378 (11)	0.0333 (10)	0.0296 (10)	0.0197 (9)	−0.0047 (8)	−0.0011 (8)
C2	0.0485 (13)	0.0355 (11)	0.0410 (11)	0.0236 (10)	−0.0105 (10)	−0.0018 (9)
C3	0.0484 (13)	0.0383 (11)	0.0400 (12)	0.0260 (10)	−0.0104 (10)	−0.0042 (9)
C4	0.0435 (12)	0.0381 (11)	0.0391 (11)	0.0220 (10)	−0.0072 (9)	0.0003 (9)
C5	0.0566 (15)	0.0428 (13)	0.0455 (13)	0.0264 (12)	−0.0154 (11)	−0.0030 (10)
C6	0.0565 (16)	0.0552 (15)	0.0552 (15)	0.0298 (13)	−0.0139 (12)	0.0118 (12)
C7	0.0628 (17)	0.0456 (14)	0.0621 (17)	0.0333 (13)	−0.0067 (14)	0.0098 (12)
C8	0.0432 (13)	0.0411 (12)	0.0607 (15)	0.0229 (11)	−0.0098 (11)	−0.0137 (11)
C9	0.0528 (15)	0.0400 (13)	0.0625 (17)	0.0243 (12)	−0.0142 (12)	−0.0133 (11)
C10	0.0483 (14)	0.0447 (13)	0.0571 (15)	0.0242 (11)	−0.0070 (11)	−0.0108 (11)
C11	0.049 (4)	0.046 (2)	0.054 (3)	0.022 (2)	−0.009 (3)	−0.009 (2)
C11A	0.025 (9)	0.058 (6)	0.069 (15)	0.019 (6)	−0.010 (8)	−0.028 (8)
C12	0.073 (5)	0.065 (4)	0.069 (5)	0.008 (4)	−0.025 (4)	−0.012 (4)
C12A	0.090 (17)	0.054 (8)	0.083 (15)	0.014 (8)	−0.008 (10)	−0.023 (8)
C13	0.055 (3)	0.074 (5)	0.070 (3)	0.003 (3)	−0.021 (2)	−0.018 (3)
C13A	0.063 (11)	0.051 (7)	0.074 (15)	0.027 (7)	−0.002 (9)	−0.016 (7)
C14	0.058 (3)	0.055 (3)	0.073 (5)	0.003 (2)	−0.002 (3)	−0.018 (3)
C14A	0.061 (12)	0.026 (7)	0.096 (15)	−0.011 (6)	−0.021 (10)	−0.002 (7)

### Geometric parameters (Å, º)

**Table:**

Eu1—O1^i^	2.4868 (16)	C3—C4	1.451 (3)
Eu1—O1^ii^	2.4868 (16)	C4—C5	1.361 (3)
Eu1—O1	2.4868 (16)	C5—H5	0.9300
Eu1—O2^ii^	2.5113 (16)	C5—C6	1.417 (4)
Eu1—O2	2.5112 (16)	C6—H6	0.9300
Eu1—O2^i^	2.5113 (16)	C6—C7	1.340 (4)
Eu1—O3^i^	2.3996 (15)	C7—H7	0.9300
Eu1—O3^ii^	2.3997 (15)	C8—C9	1.471 (3)
Eu1—O3	2.3997 (15)	C9—H9	0.9300
S1—C4	1.716 (2)	C9—C10	1.318 (4)
S1—C7	1.703 (3)	C10—H10	0.9300
S2—C11	1.715 (9)	C10—H10A	0.9300
S2—C14	1.710 (7)	C10—C11	1.443 (6)
S2A—C11A	1.72 (3)	C10—C11A	1.452 (17)
S2A—C14A	1.70 (2)	C11—C12	1.322 (14)
O1—C1	1.266 (3)	C11A—C12A	1.29 (3)
O2—O4^iii^	11.564 (3)	C12—H12	0.9300
O2—C1	1.266 (3)	C12—C13	1.446 (15)
O3—H3A	0.818 (13)	C12A—H12A	0.9300
O3—H3B	0.826 (13)	C12A—C13A	1.47 (3)
O4—H4	0.836 (14)	C13—H13	0.9300
O4—C8	1.305 (3)	C13—C14	1.358 (9)
O5—C8	1.203 (3)	C13A—H13A	0.9300
C1—C2	1.466 (3)	C13A—C14A	1.34 (2)
C2—H2	0.9300	C14—H14	0.9300
C2—C3	1.315 (3)	C14A—H14A	0.9300
C3—H3	0.9300		
			
O1^i^—Eu1—O1^ii^	76.88 (6)	C2—C3—H3	116.7
O1^i^—Eu1—O1	76.88 (6)	C2—C3—C4	126.5 (2)
O1^ii^—Eu1—O1	76.88 (6)	C4—C3—H3	116.7
O1—Eu1—O2^ii^	79.32 (6)	C3—C4—S1	123.01 (17)
O1—Eu1—O2^i^	126.87 (6)	C5—C4—S1	110.47 (18)
O1^ii^—Eu1—O2	126.87 (6)	C5—C4—C3	126.4 (2)
O1^i^—Eu1—O2^i^	51.62 (5)	C4—C5—H5	123.6
O1^ii^—Eu1—O2^ii^	51.62 (5)	C4—C5—C6	112.8 (2)
O1^ii^—Eu1—O2^i^	79.33 (6)	C6—C5—H5	123.6
O1—Eu1—O2	51.62 (5)	C5—C6—H6	123.6
O1^i^—Eu1—O2	79.33 (6)	C7—C6—C5	112.8 (2)
O1^i^—Eu1—O2^ii^	126.87 (6)	C7—C6—H6	123.6
O2^ii^—Eu1—O2	119.037 (14)	S1—C7—H7	124.1
O2^i^—Eu1—O2	119.040 (14)	C6—C7—S1	111.7 (2)
O2^i^—Eu1—O2^ii^	119.038 (14)	C6—C7—H7	124.1
O3^i^—Eu1—O1	157.80 (6)	O4—C8—C9	112.8 (2)
O3^i^—Eu1—O1^ii^	91.68 (6)	O5—C8—O4	123.0 (2)
O3^ii^—Eu1—O1^ii^	119.45 (5)	O5—C8—C9	124.1 (3)
O3^ii^—Eu1—O1^i^	157.80 (6)	C8—C9—H9	119.6
O3—Eu1—O1^ii^	157.80 (6)	C10—C9—C8	120.7 (3)
O3^ii^—Eu1—O1	91.68 (6)	C10—C9—H9	119.7
O3—Eu1—O1	119.45 (5)	C9—C10—H10	116.2
O3—Eu1—O1^i^	91.68 (6)	C9—C10—H10A	119.2
O3^i^—Eu1—O1^i^	119.45 (5)	C9—C10—C11	127.5 (5)
O3—Eu1—O2^ii^	141.12 (6)	C9—C10—C11A	121.6 (13)
O3—Eu1—O2	67.86 (5)	C11—C10—H10	116.2
O3^ii^—Eu1—O2^ii^	67.86 (5)	C11A—C10—H10A	119.2
O3^ii^—Eu1—O2^i^	141.12 (6)	C10—C11—S2	122.5 (7)
O3^i^—Eu1—O2	141.12 (6)	C12—C11—S2	111.9 (8)
O3^i^—Eu1—O2^i^	67.86 (5)	C12—C11—C10	125.6 (10)
O3^ii^—Eu1—O2	78.67 (7)	C10—C11A—S2A	117 (2)
O3^i^—Eu1—O2^ii^	78.67 (7)	C12A—C11A—S2A	111.6 (16)
O3—Eu1—O2^i^	78.67 (7)	C12A—C11A—C10	131 (3)
O3^i^—Eu1—O3^ii^	77.30 (7)	C11—C12—H12	123.7
O3—Eu1—O3^ii^	77.30 (7)	C11—C12—C13	112.6 (12)
O3^i^—Eu1—O3	77.30 (7)	C13—C12—H12	123.7
C7—S1—C4	92.19 (13)	C11A—C12A—H12A	122.7
C14—S2—C11	92.3 (5)	C11A—C12A—C13A	115 (3)
C14A—S2A—C11A	91.3 (14)	C13A—C12A—H12A	122.7
C1—O1—Eu1	95.50 (12)	C12—C13—H13	123.9
Eu1—O2—O4^iii^	150.31 (5)	C14—C13—C12	112.2 (8)
C1—O2—Eu1	94.35 (13)	C14—C13—H13	123.9
C1—O2—O4^iii^	109.24 (13)	O3^ii^—C13A—H13A	143.0
Eu1—O3—H3A	119 (2)	C12A—C13A—H13A	125.4
Eu1—O3—H3B	122 (2)	C14A—C13A—C12A	109 (2)
H3A—O3—H3B	113 (3)	C14A—C13A—H13A	125.4
C8—O4—H4	112 (3)	S2—C14—H14	124.6
O1—C1—C2	122.4 (2)	C13—C14—S2	110.8 (6)
O2—C1—O1	118.50 (19)	C13—C14—H14	124.6
O2—C1—C2	119.0 (2)	S2A—C14A—H14A	123.3
C1—C2—H2	117.9	C13A—C14A—S2A	113 (2)
C3—C2—C1	124.3 (2)	C13A—C14A—H14A	123.3
C3—C2—H2	117.9		
			
Eu1—O1—C1—O2	−1.5 (2)	C7—S1—C4—C3	175.6 (2)
Eu1—O1—C1—C2	176.2 (2)	C7—S1—C4—C5	−0.3 (2)
Eu1—O2—C1—O1	1.5 (2)	C8—C9—C10—C11	−176.2 (4)
Eu1—O2—C1—C2	−176.33 (19)	C8—C9—C10—C11A	−175.8 (6)
S1—C4—C5—C6	0.0 (3)	C9—C10—C11—S2	7.3 (6)
S2—C11—C12—C13	−4.1 (14)	C9—C10—C11—C12	−172.7 (10)
S2A—C11A—C12A—C13A	0.4 (6)	C9—C10—C11A—S2A	−169.2 (7)
O1—C1—C2—C3	6.8 (4)	C9—C10—C11A—C12A	11.0 (8)
O2—C1—C2—C3	−175.5 (3)	C10—C11—C12—C13	175.8 (6)
O3^ii^—C13A—C14A—S2A	−5.5 (5)	C10—C11A—C12A—C13A	−179.8 (3)
O4^iii^—O2—C1—O1	163.18 (17)	C11—S2—C14—C13	−1.1 (6)
O4^iii^—O2—C1—C2	−14.6 (2)	C11—C12—C13—C14	3.4 (14)
O4—C8—C9—C10	177.6 (3)	C11A—S2A—C14A—C13A	−0.2 (5)
O5—C8—C9—C10	0.6 (5)	C11A—C12A—C13A—O3^ii^	18.1 (16)
C1—C2—C3—C4	−175.5 (2)	C11A—C12A—C13A—C14A	−0.5 (8)
C2—C3—C4—S1	6.9 (4)	C12—C13—C14—S2	−1.0 (9)
C2—C3—C4—C5	−177.8 (3)	C12A—C13A—C14A—S2A	0.5 (7)
C3—C4—C5—C6	−175.7 (3)	C14—S2—C11—C10	−176.9 (4)
C4—S1—C7—C6	0.6 (3)	C14—S2—C11—C12	3.1 (9)
C4—C5—C6—C7	0.4 (4)	C14A—S2A—C11A—C10	−179.9 (2)
C5—C6—C7—S1	−0.7 (4)	C14A—S2A—C11A—C12A	−0.1 (4)

Symmetry codes: (i) −*x*+*y*, −*x*+1, *z*; (ii) −*y*+1, *x*−*y*+1, *z*; (iii) −*y*+2/3, *x*−*y*+1/3, *z*−2/3.

### Hydrogen-bond geometry (Å, º)

*Cg*1 is the centroid of the S1/C4–C7 ring.

**Table:**

*D*—H···*A*	*D*—H	H···*A*	*D*···*A*	*D*—H···*A*
O3—H3*A*···O5	0.82 (1)	1.94 (2)	2.729 (3)	162 (3)
O3—H3*B*···O1^iv^	0.82 (1)	1.89 (2)	2.693 (2)	165 (3)
O4—H4···O2	0.84 (1)	1.78 (2)	2.614 (3)	177 (4)
C7—H7···*Cg*1^v^	0.93	3.10	3.869 (3)	141

Symmetry codes: (iv) −*x*+*y*, −*x*+1, *z*−1; (v) −*x*+*y*−2/3, −*x*−4/3, *z*−1/3.
